# Differences in quantitative myocardial perfusion mapping by CMR at 1.5 T and 3 T

**DOI:** 10.1016/j.ahjo.2024.100388

**Published:** 2024-03-23

**Authors:** George R. Abraham, Colin Berry, Qing Fu, Stephen P. Hoole, Jonathan R. Weir-McCall

**Affiliations:** a Royal Papworth Hospital NHS Foundation Trust, Papworth Road, Cambridge Biomedical Campus, Cambridge CB2 0AY, United Kingdom of Great Britain and Northern Ireland; b University of Cambridge, The Old Schools, Trinity Lane, Cambridge CB2 1TN, United Kingdom of Great Britain and Northern Ireland; c NHS Greater Glasgow and Clyde Health Board, Gartnavel Royal Hospital Campus, 1055 Great Western Road, Glasgow G12 0XH, United Kingdom of Great Britain and Northern Ireland; d British Heart Foundation Glasgow Cardiovascular Research Centre, University of Glasgow, BHF Glasgow Cardiovascular Research Centre (GCRC), 126 University Place, Glasgow G12 8TA, United Kingdom of Great Britain and Northern Ireland; e Department of Radiology, Union Hospital, Tongji Medical College, Huazhong University of Science and Technology, Wuhan, China

**Keywords:** Cardiac magnetic resonance imaging, Quantitative perfusion imaging, Coronary microvascular dysfunction

Quantitative myocardial perfusion mapping by cardiac magnetic resonance imaging (CMR) is an emerging non-invasive diagnostic tool for coronary heart disease. Advantages of quantitative analysis over traditional qualitative assessment include the objective assessment of myocardial blood flow in conditions of globally reduced flow such as coronary microvascular dysfunction [1]. Normal values for stress myocardial blood flow (MBF) and myocardial perfusion reserve (MPR) have been established [2,3] and have been used for diagnostic validation, for example, against invasive functional tests for coronary microvascular dysfunction [4]. Thresholds have also been proposed for determining if a stress test is adequate (stress MBF >1.43 ml/g/min) [5] and whether there is obstructive coronary artery disease (stress MBF <1.94 ml/g/min) [6]. It is unclear however, whether field strength (3 T vs 1.5 T) has a significant impact on flow quantitation, and whether adjustments to the thresholds are required depending on the type of scanner used.

We performed a retrospective analysis of 212 stress perfusion CMR scans comparing global stress and resting MBF, stress endocardial:epicardial ratio and MPR. Scans were performed according to standard protocol at 1.5 T (Aera) in 102 patients and 3.0 T (Prisma, Siemens, Erlangen, Germany) in 110 patients. Basal, mid-ventricular and apical short axis perfusion images were acquired during hyperaemia induced by peripheral venous infusion of adenosine (140–210 μg/kg/min), and at rest. Automated inline perfusion mapping was generated by the dual sequence technique described by Kellman et al. [1] Univariate adjustment was perfomed for likely confounders: gender, rate-pressure product and diagnosis (IBM SPSS v.27). Diagnosis was coded for each scan as either: normal, ischemia with no obstructive coronary disease (INOCA), epicardial coronary artery disease (CAD) with ischemia, CAD without ischemia, and non-ischemic cardiomyopathy.

Resting MBF was higher at 1.5 T versus 3 T after adjustment for baseline rate-pressure product, gender and cardiac diagnosis (0.70 ± 0.28 vs. 0.59 ± 0.35 ml/g/min, *p* = 0.01). Mean stress MBF was also higher at 1.5 T versus 3 T albeit not statistically significant (stress MBF: 1.81 ± 0.72 ml/g/min vs. 1.64 ± 0.70 ml/g/min, *p* = 0.09). Endocardial:epicardial ratio (0.87 ± 0.15 vs. 0.88 ± 0.12, *p* = 0.76) and MPR (2.73 ± 1.06 vs. 2.92 ± 0.92, *p* = 0.18) were not significantly different at 1.5 T versus 3 T.

Amongst the 77 scans coded as ‘normal’, there were differences in both resting MBF (1.5 T: 0.79 ± 0.35 ml/g/min vs. 3 T: 0.60 ± 0.17 ml/g/min, *p* < 0.01) and stress MBF (1.5 T: 2.27 ± 0.77 ml/g/min vs. 3 T: 1.90 ± 0.66 ml/g/min, *p* = 0.02). MPR and endocardial: epicardial ratio values were no different between field strengths (Fig. 1).

**Fig. 1:**
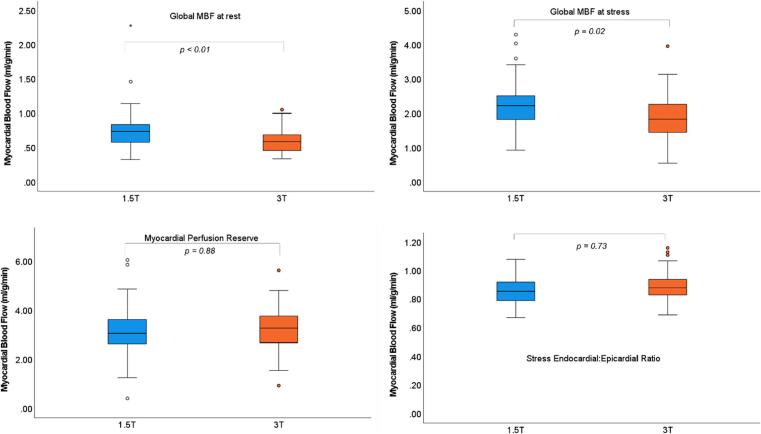
Box-plots show data obtained from ‘normal’ scans. Comparison of means (independent samples *t*-test) revealed significant differences in absolute MBF values at rest and stress, however ratio variables were no different between field strengths.

Our results indicate myocardial blood flow may differ when assessed at different MRI field strengths, with stress MBF 19 % higher at 1.5 T even after adjustment for gender and hemodynamic status. While this is most likely related to field strength, it may be due to the pulse sequence used, as there was a staggered transition from FLASH to SSFP sequence readouts on the 3 T scanner during the study period. Further investigation to determine scanner and/or sequence specific reference ranges for myocardial blood flow quantification may be necessary before routine clinical use.

In contrast, the use of quantitative ratios (MPR and endocardial:epicardial ratio) is transferrable across field strengths and may be more immediately useful for current clinical investigation and diagnosis.

## CRediT authorship contribution statement

**George R. Abraham:** Writing – original draft, Visualization, Methodology, Formal analysis, Data curation, Conceptualization. **Colin Berry:** Writing – review & editing, Supervision, Resources. **Qing Fu:** Writing – review & editing, Data curation. **Stephen P. Hoole:** Writing – review & editing, Supervision, Resources. **Jonathan R. Weir-McCall:** Writing – review & editing, Supervision, Methodology, Investigation, Formal analysis, Data curation, Conceptualization.

## Declaration of competing interest

CB is employed by the University of Glasgow which holds consultancy and research agreements for his work with Abbott Vascular, AstraZeneca, Boehringer Ingelheim, Coroventis, HeartFlow, Menarini, MSD, Novartis, Servier, Siemens Healthcare, TherOx, Inc. and Valo Health and receives research funding from the British Heart Foundation grant (RE/18/6134217, BHF/FS/17/26/32744, PG/19/28/34310), Chief Scientist Office, EPSRC (EP/R511705/1, EP/S030875/1), European Union (754946-2), Medical Research Council (MR/S018905/1) and UKRI (MC/PC/20014). All other authors declare no competing interests relevant to this work.

## Appendix A Supplementary data

Supplementary material Image 1
